# Recent Advances in Genome Maintenance Processes

**DOI:** 10.3390/ijms25105131

**Published:** 2024-05-09

**Authors:** Ingrid Tessmer

**Affiliations:** Rudolf Virchow Center for Integrative and Translational Bioimaging, University of Würzburg, 97080 Würzburg, Germany; ingrid.tessmer@uni-wuerzburg.de

Given life’s dependence on genome maintenance, unsurprisingly, investigations of the molecular processes involved in protecting the genome or, failing this, repairing damages to and alterations introduced into genetic material are at the forefront of current research. In recent years, increasing insights into the intricate interplay between proteins and DNA that safeguard our genetic code have emerged, including the discovery of novel protein factors, protein functions, and interactions between different DNA processing systems. The contributions assembled in this Special Issue address different types of DNA damage repair, DNA metabolism regulation, and DNA compaction. In addition to novel findings on key protein systems involved in genome maintenance and their relevance to disease, state-of-the-art ensemble and single-molecule methodologies for studying their interactions with (damaged) DNA in vitro and in vivo are discussed.

One central factor in DNA repair and metabolic processes such as DNA replication is replication protein A (RPA) [1,2,3]. RPA coats single-stranded DNA (ssDNA) stretches that emerge during DNA metabolism with high affinity, protecting them from nucleophilic attack and other causes of DNA damage. In addition, RPA also plays an essential role in mediating the recruitment of different proteins required for DNA processing, for example, during DNA replication or repair. Heinz Peter Nasheuer and colleagues (Nasheuer et al.) provide a comprehensive overview of the current knowledge on the structure, function, and interactions of RPA in DNA damage response and replication, as well as the roles of RPA and an alternative form of RPA in neurodegenerative diseases and cancer.

Multiple types of exogenous (such as radiation or chemotherapeutics) and endogenous agents (such as reactive oxygen species or alkylating metabolic products) result in different types of damages in DNA. For instance, high-energy radiation can completely sever the backbones of both strands in double-stranded DNA, leading to double-strand breaks (DSBs). Unrepaired DSBs are lethal to cells; meanwhile, their inaccurate repair can cause genetic instability due to chromosomal deletions or translocations [4,5]. Two major DNA repair pathways that target and repair DSBs are known: homologous recombination (HR, which uses the sister chromatid DNA as the template for repair), which is highly accurate, and non-homologous end joining (NHEJ, which merely ties together broken DNA ends without sequence control), which is less accurate. The contributions of a third, less well-known DSB repair pathway, microhomology-mediated end joining (MMEJ), to genome instability are summarised in the review by Yuning Jiang, which also discusses the potential to target this highly inaccurate DSB repair pathway in cancer therapy.

Subtle chemical modifications of DNA bases caused by various damaging agents can also be extremely dangerous for cells, as they can either present replication blocks or induce base mispairing during DNA replication, leading to mutagenesis [6,7]. Spontaneous deamination of cytosine, for example, generates uracil, which miscodes for adenine during replication, leading to C > T transition mutations [8]. The uracil hence needs to be removed from the DNA. Uracil excision is performed by the uracil DNA N-glycosylase (UNG), one of several different DNA glycosylases that each target different types of chemical base modifications and initiate the base excision repair (BER) cascade. In their contribution of a research article to this collection, Dmitry Zharkov and colleagues (Diatlova et al.) investigate the unique function of a viral UNG in DNA replication as a processivity factor.

Among the most highly mutagenic DNA lesions are those caused by the alkylation of specific oxygen atoms in DNA bases (O-alkylation, at the O^6^ position of guanine and the O^4^ position of thymine). Their repair is handled by the dedicated direct damage reversal mechanism of the DNA alkyltransferase protein family. This family of proteins, their evolution, function, and interactions, as well as the current knowledge on approaches to their inhibition during O^6^-alkylating agent chemotherapy, are discussed in the review by Ingrid Tessmer and Geoffrey Margison.

The only other (currently known) direct damage reversal system in humans is that of the dioxygenase AlkB family enzymes, responsible for the repair of distinct N-alkylation products in DNA. In this Special Issue, Lyubov Kanazhevskaya, Vladimir Koval, and colleagues (Kanazhevskaya et al.) present novel structural and kinetic investigations of single-stranded DNA damage binding and processing by the poorly understood alkylation repair enzyme ALKBH3.

In contrast to these subtle chemical base modifications, the nucleotide excision repair (NER) system targets bulky adducts and strongly DNA helix-distorting lesions. Sripriya Raja and Bennett van Houten detail recent studies from the van Houten group on an NER lesion recognition factor, the UV-damaged DNA binding (UV-DDB) protein complex. In their work, they revealed functions of UV-DDB in lesion sensing for base excision repair (BER) and in chromatin decompaction, which provides access for BER proteins to repair DNA damages in the chromatin context.

Chromatin decompaction is thus essential not only for gene transcription [9,10] and replication [11] but also for efficient DNA repair. Because lesions in DNA that is wrapped tightly around nucleosomes are often difficult to process by DNA repair machineries, defects in chromatin structure regulation are associated with genomic instability. Nucleosomal DNA unwrapping is mediated by chromatin remodeling enzymes, as well as post-translational modifications (PTMs) to histones (in particular, in histone tails). Kathiresan Selvam, John Wyrick, and Michael Parra elucidate our current understanding of the diverse histone PTMs that modulate the accessibility of DNA lesions in chromatin to repair proteins, or histone interactions with chromatin remodelers and DNA repair proteins, and discuss pathological histone mutations, such as those seen in cancers.

In the cellular context, chromosomal compaction and DNA metabolism are affected by the crowded environment. Crowding is further locally enhanced in cells by membrane-enclosed compartmentalisation and, in addition, by the formation of membraneless biomolecular condensates, which have attracted profound interest since their initial description in 2017 [12]. They have been shown to play a role in modulating gene transcription and DNA repair in cells [13,14,15,16] and have also been exploited in biotechnological approaches [17,18]. Dylan Collette, David Dunlap, and Laura Finzi address the effects of different crowding agents on DNA compaction, dynamics, and interactions. Our understanding of protein mechanisms at the molecular level is largely owed to in vitro experiments. While these can be conducted in the presence of precisely controlled concentrations of select macromolecular crowding agents to mimic the cellular environment, this review highlights the need to understand and consider their effects on the investigated systems when comparing in vitro and in vivo studies.
